# Coating with luminal gut-constituents alters adherence of nanoparticles to intestinal epithelial cells

**DOI:** 10.3762/bjnano.5.239

**Published:** 2014-12-02

**Authors:** Heike Sinnecker, Katrin Ramaker, Andreas Frey

**Affiliations:** 1 Division of Mucosal Immunology & Diagnostics, Priority Program Asthma & Allergy, Research Center Borstel, Leibniz Center for Medicine and Biosciences, Parkallee 22, 23845 Borstel, Germany, Member of the German Center for Lung Research

**Keywords:** adherence, agglomeration, intestinal epithelial cells, nanoparticles (NPs), protein

## Abstract

**Background:** Anthropogenic nanoparticles (NPs) have found their way into many goods of everyday life. Inhalation, ingestion and skin contact are potential routes for NPs to enter the body. In particular the digestive tract with its huge absorptive surface area provides a prime gateway for NP uptake. Considering that NPs are covered by luminal gut-constituents en route through the gastrointestinal tract, we wanted to know if such modifications have an influence on the interaction between NPs and enterocytes.

**Results:** We investigated the consequences of a treatment with various luminal gut-constituents on the adherence of nanoparticles to intestinal epithelial cells. Carboxylated polystyrene particles 20, 100 and 200 nm in size represented our anthropogenic NPs, and differentiated Caco-2 cells served as model for mature enterocytes of the small intestine. Pretreatment with the proteins BSA and casein consistently reduced the adherence of all NPs to the cultured enterocytes, while incubation of NPs with meat extract had no obvious effect on particle adherence. In contrast, contact with intestinal fluid appeared to increase the particle-cell interaction of 20 and 100 nm NPs.

**Conclusion:** Luminal gut-constituents may both attenuate and augment the adherence of NPs to cell surfaces. These effects appear to be dependent on the particle size as well as on the type of interacting protein. While some proteins will rather passivate particles towards cell attachment, possibly by increasing colloid stability or camouflaging attachment sites, certain components of intestinal fluid are capable to modify particle surfaces in such a way that interactions with cellular surface structures result in an increased binding.

## Introduction

Anthropogenic nanoparticles (NPs) are incorporated into a variety of consumer products to improve their function, prolong their shelf life or protect them against pathogenic microbes [1–2]. Besides that NPs are also involuntarily released by humans via industrial production, manufacturing and combustion processes. This inevitably raises the question what happens if these NPs get in touch with the human body, be it by inhalation, ingestion or skin contact. Consequences of anthropogenic particles in dusts and exhaust gases caused by industry and road traffic to humans and to the environment have been of interest for some time [3–5]. Also, numerous in vitro, less in vivo studies and some case reports described adverse effects caused by NPs on cell viability, protein functions and DNA stability as well as other cell and tissue impairments [6–10]. In the light of this, a definite assessment of the health risk to humans by NPs is demanded more and more [11].

Among the different routes of entry for potentially dangerous NPs, the human digestive tract with its huge surface area, primarily responsible for the resorption of nutrients, offers an attractive gateway for orally taken-up NPs to traverse into the organism. However, thorough investigations of the actual fate of NPs within the digestive tract are limited. The behavior of NPs after ingestion is certainly influenced by many factors such as dietary status, level of mucosal and enzymatic secretions, different pH-values, gastrointestinal transit time and the intestinal microflora [12]. Important players on the gastrointestinal scene which must be taken into consideration when investigating NPs in the gut are the various constituents of nutrition and digestive fluids. As it has been shown that the efficiency of particle uptake after oral challenge is a particle size-dependent event, with smaller particles being taken up more frequently than larger ones [13–15], a possible protein-induced particle agglomeration must be considered an issue when looking into potential translocation. Besides a conceivable increase in particle size, the modification of the particle surface with a protein corona will influence the interaction of NPs with the intestinal mucosal defense system - a multifactorial barrier against mucosal intruders, composed of, e.g., enzymes, soluble surrogate receptors, immunoglobulins and secreted mucus [16]. As a final barrier the enterocyte cell layer must be breached. These cells are columnar epithelial cells with a brush border membrane on the apical side, covered by the glycocalyx, a dense mesh of glycostructures [17], and connected to each other by tight junctions [18]. From all this it follows that it probably depends on a number of different factors to what extent inadvertently swallowed particulate matter actually will traverse into the body. In our study, we focused on just one aspect, namely the effect which the different protein components that ingested NPs encounter in the intestinal tract may have on particle adherence to epithelial cells. The study using an in vitro cell culture as model system was intended to reveal at least some initial information about this very complex situation.

## Results and Discussion

**Figure 1:**
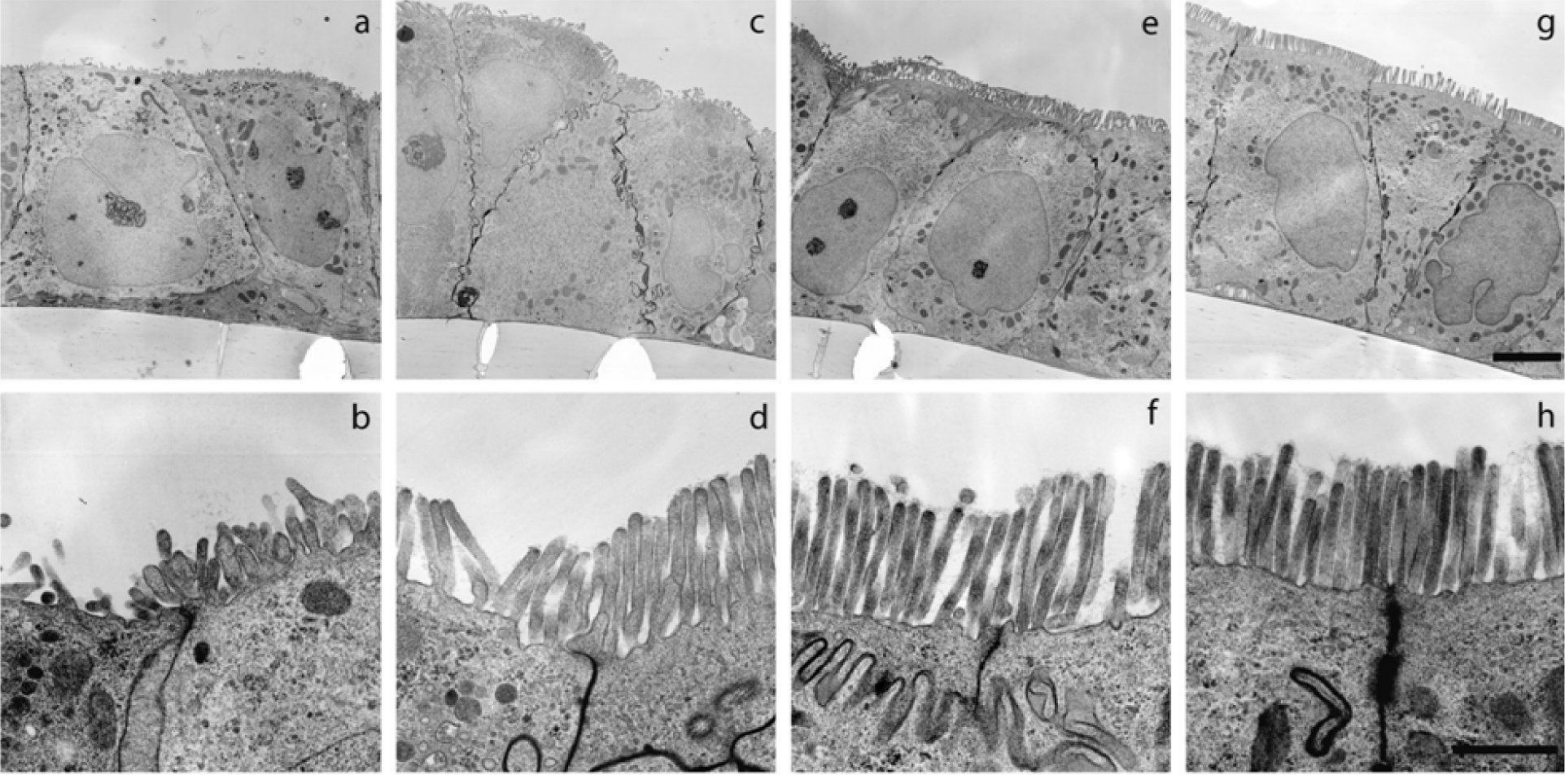
Differentiation of Caco-2 cells over 21 days post-confluence. a,b: With reaching confluence, cells have built a monolayer and rudimentary microvilli are visible; c,d: 7 days after formation of a confluent monolayer microvilli are pronounced; e,f: 14 days post-confluence cells are grown to a columnar epithelium, the microvilli have increased, tight junctions are formed and the glycocalyx between and on the top of the microvilli is visible; g,h: 21 days post-confluence all structural attributes of intestinal epithelial cells are formed. Upper panel (a,c,e,g): Scale bar, 5 µm, lower panel (b,d,f,h): Scale bar, 1 µm.

To investigate the interaction of NPs with intestinal epithelial cells we used the human colorectal adenocarcinoma cell line Caco-2 as model enterocytes because Caco-2 cells can spontaneously differentiate into a columnar epithelium after reaching confluence [19] and because the morphological and biochemical features of differentiated Caco-2 cells are highly comparable to those of mature enterocytes of the small intestine [20–21]. Electron microscopic analysis was used to monitor the differentiation of Caco-2 cells over 21 days (Figure 1). After 14 days post-confluence the relevant structural features of enterocytes were well-developed: columnar cells with brush border microvilli and a glycocalyx on the apical side. Therefore, we used the differentiated cells at this point in time for our interaction studies of NPs with intestinal epithelial cells.

**Figure 2:**
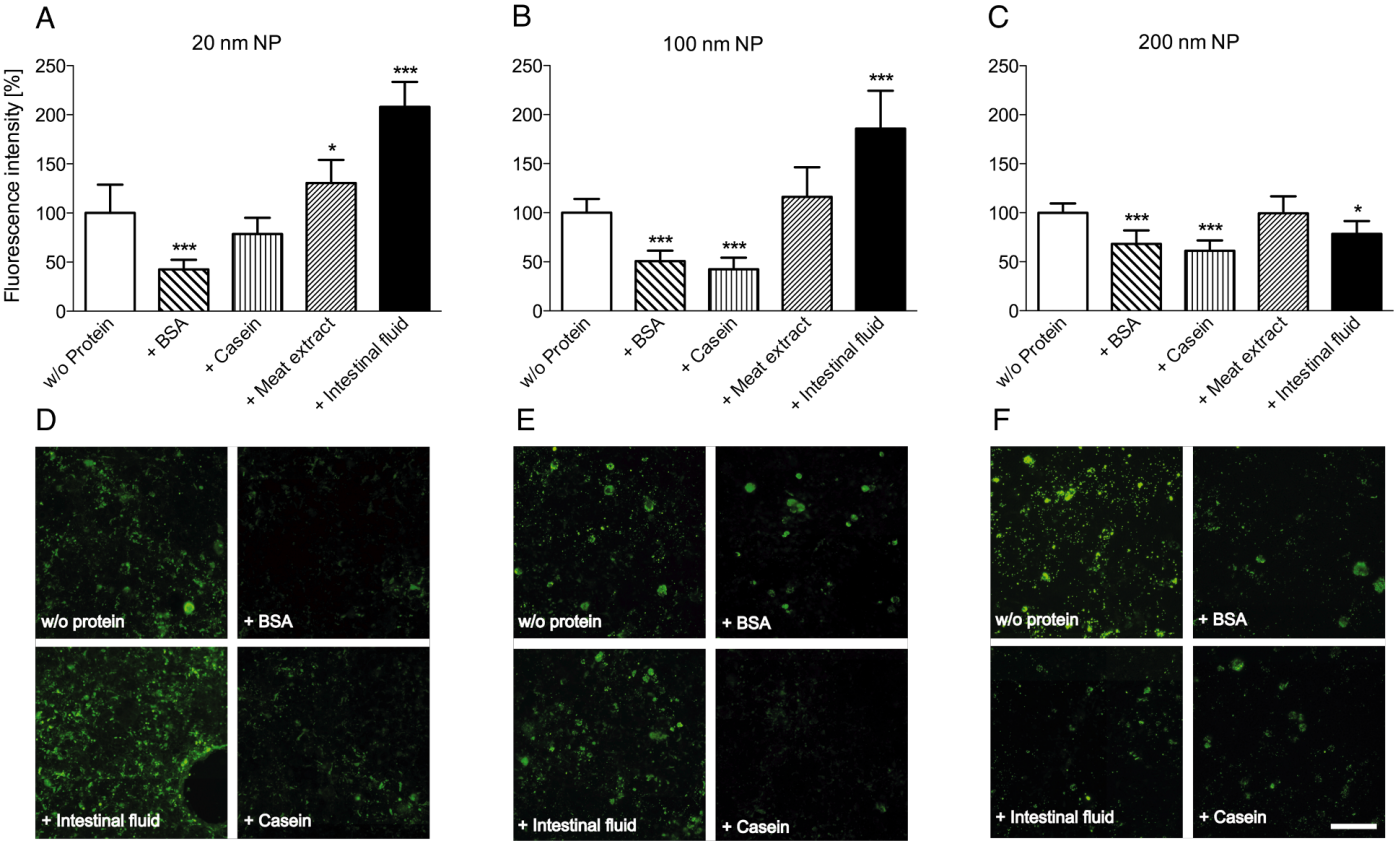
Interaction of fluorescent NPs with Caco-2 cells in the presence of proteinaceous compounds. Differently sized NPs, without or with pretreatment with different protein mixtures, were incubated with Caco-2 cells. After extensive washing, particle adherence to and/or uptake by the cells was determined by fluorescence microscopy. Values were normalized to intensities obtained with untreated particles (A, B, C; mean + SD of 6–8 individual experiments; *p* < 0.05 (*) and *p* < 0.001 (***), ANOVA with Dunnett’s post-hoc test). En-face fluorescence microscope images (D, E, F) show variations in NP adherence, depending on the pretreatment with proteins. Scale bar, 100 µm.

As model anthropogenic nanoparticles, surface-carboxylated fluorescent polystyrene particles of different size (20, 100 and 200 nm) were used. These particles were incubated with various proteinaceous compounds to simulate different gut environments, and afterwards the interaction of these pretreated NPs with cultured Caco-2 cells was investigated. Particle adherence to or uptake by the cells was analyzed via fluorescence microscopy (Figure 2). Quantitative results were obtained by comparing fluorescence intensities using image analysis software, whereby values obtained with untreated NPs (control without protein pretreatment) were set to 100% fluorescence intensity. We found that, in the presence of the proteins bovine serum albumin (BSA) and casein the fluorescence intensities were discernibly decreased, no matter of which size the NPs were. We speculate that a protein coating produced by passive adsorption of the proteins to the NPs is responsible for this observed reduction in particle adherence to the cells. The potential consequences of the presence of proteins in biological systems on the behaviour of NPs in that environment are gaining increasing interest. Indeed, the formation of a protein corona around NPs was reviewed in several articles in the last years [22–24], and possible implications for the particle–cell interactions are considered. For example, it was shown that human serum albumin (HSA), BSA and fetal bovine serum can build a protein corona around NPs whereby the particles are stabilized against agglomeration and the colloid stability of the particle suspension is improved [25–26], and that the absorption of proteins on the particle surface can obviously reduce the particle adhesion to cell membranes and the uptake by cultured cells [27–29]. To gain additional information on the NP attachment and on the effect of the proteinaceous compounds we took a closer look at different microscope images (Figure 2D–F). The fluorescence pattern obtained with untreated 20 nm and 100 nm particles – displaying distinct “spots” – indicates a certain degree of NP agglomeration under these conditions, since individual particles of 20 or 100 nm size ought not to be visible at the magnification used during light microscopy.

**Figure 3:**
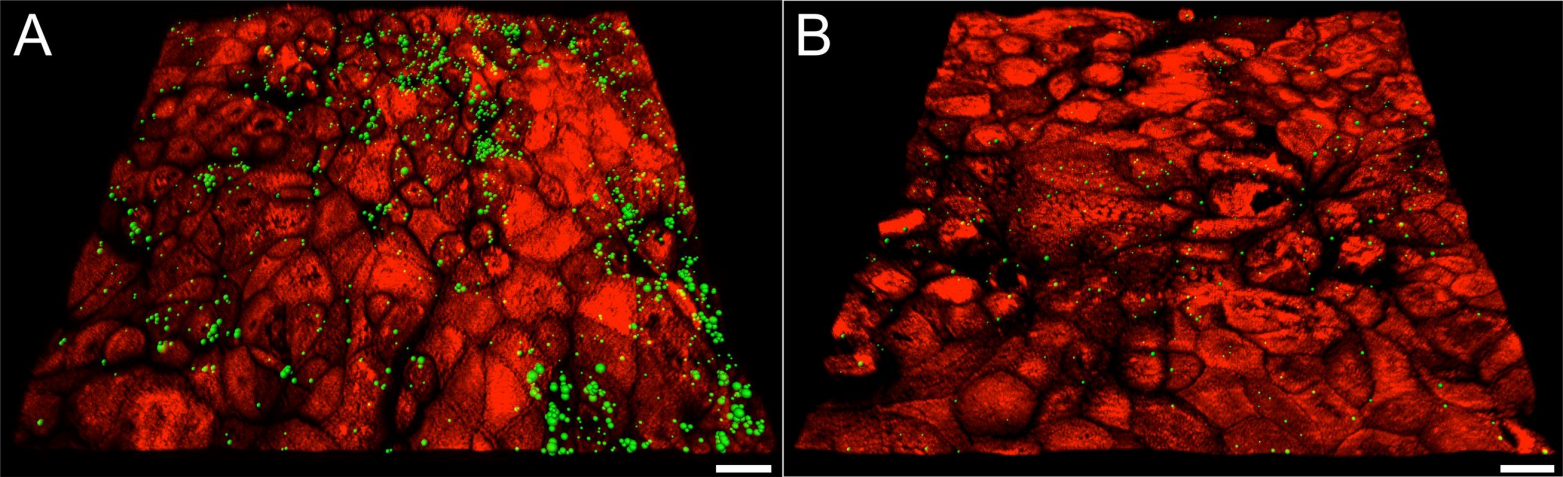
Adherence of 100 nm NPs to Caco-2 cells. The glycocalyx was counter-stained with a lectin (red) to visualize the cell surface of the monolayer. Stacks of 2-dimensional microscope images were processed with the software Imaris x64 to give a 3D rendition. A: In the absence of BSA, NPs are attached to the cell surface mainly in form of particle agglomerates (green spots in variable size). B: After preincubation of NPs with BSA only few and small particle spots are visible. Scale bar: 15 µm.

The proteins BSA and casein seem to diminish the contact of NPs with the cell surface by enhancing the NP colloid stability. This effect is particularly evident in software-processed microscope images of 100 nm particles on Caco-2 cells grown on transwell filters (Figure 3A, B; counter-staining of the cell surface with a lectin). Pretreatment with the protein BSA appears to alleviate agglomeration as well as adherence. Only few distinct spots are visible on the cell surface, compared to a pronounced adherence of markedly larger particle agglomerates in the absence of protein. It seems also that the NPs are preferably localised in the regions where cell contacts can be found and not where a pronounced glycocalyx is present. Step-wise microscopic scanning through consecutive cell “slices” from the apical to the basolateral side showed that the decrease in fluorescence at the cell surface was not associated with an increased fluorescence inside the cells, indicating that the protein coat had not led to an enhanced NP uptake by the cells.

When analyzing the effect of NP treatment with meat extract, a mixture of peptides and amino acids representative for an alimentary peptide source, we noticed that this seemed to have no substantial effect on the adherence of NPs to the cells, only with the 20 nm NPs slightly higher fluorescence intensities were measured (Figure 2A). Individual peptides and amino acids of this extract do obviously not modify the surface of the NPs in the same way as complete proteins, such as BSA.

In contrast to the treatment with individual proteins, the incubation of the smaller (20 and 100 nm) NPs in native intestinal fluid resulted in higher fluorescence intensities, i.e., higher attachment to the cells. This effect was not observed with the larger, 200 nm particles, which exhibited a slightly reduced cell binding after incubation with intestinal fluid, similar to the result obtained by treatment with individual proteins.

**Figure 4:**
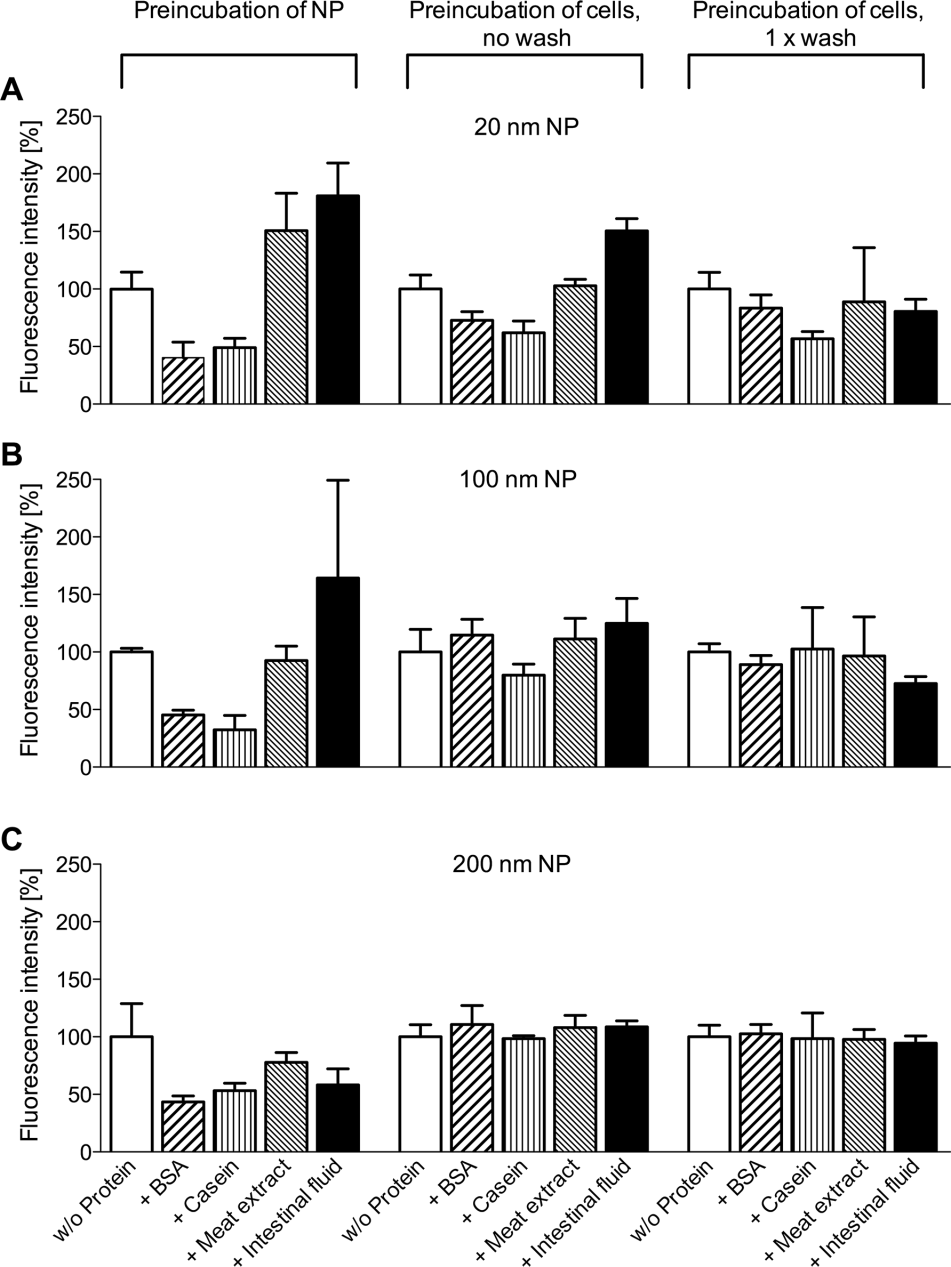
Interaction of fluorescent NPs with cells after preincubation with proteinaceous compounds. Either differently sized NPs or Caco-2 cells were preincubated with buffer or with different proteins before interacting with each other. Particle adherence to the cells was determined by fluorescence microscopy. Values were normalized to intensities obtained with untreated cells and particles (mean + SD of three experiments).

In order to verify if the observed effects were due to a modification of the NPs by the different proteins or if the proteins and luminal gut-constituents had in fact modified the surface of the cells and thereby evoked the different NP adherence patterns, we performed another set of experiments where the cells were first treated with the various proteinaceous compounds. Only after removal of the proteins – and a facultative washing step –, non-treated, “naked” NPs in buffer were added, incubated with the cells, and the fluorescence of bound particles was determined as before (Figure 4).

In this setting, the effects of the proteins on NP attachment were visibly reduced. Indeed, for the 200 nm NPs (Figure 4C) virtually no differences in the particle adherence were observed between the control cells (without protein preincubation) and the cells preincubated with the various proteinaceous compounds. For the 100 nm NPs (Figure 4B), a small decrease in particle attachment was found after treatment of the cells with casein, while intestinal fluid treatment slightly enhanced particle attachment. The most pronounced, though not statistically significant changes were seen with the 20 nm NPs where the observed effects paralleled the ones obtained by preincubation of the particles with the proteins (Figure 4A). As the additional washing step tended to push the attachment pattern in the direction of non-treated cells and particles, it seems conclusive that the main interaction occurred indeed between particles and proteins and not between proteins and cells. Small amounts of proteins which remain on the cell surface after cell treatment may still interact with added NPs and influence their adherence behavior, but in general particle pretreatment has a higher impact.

**Figure 5:**
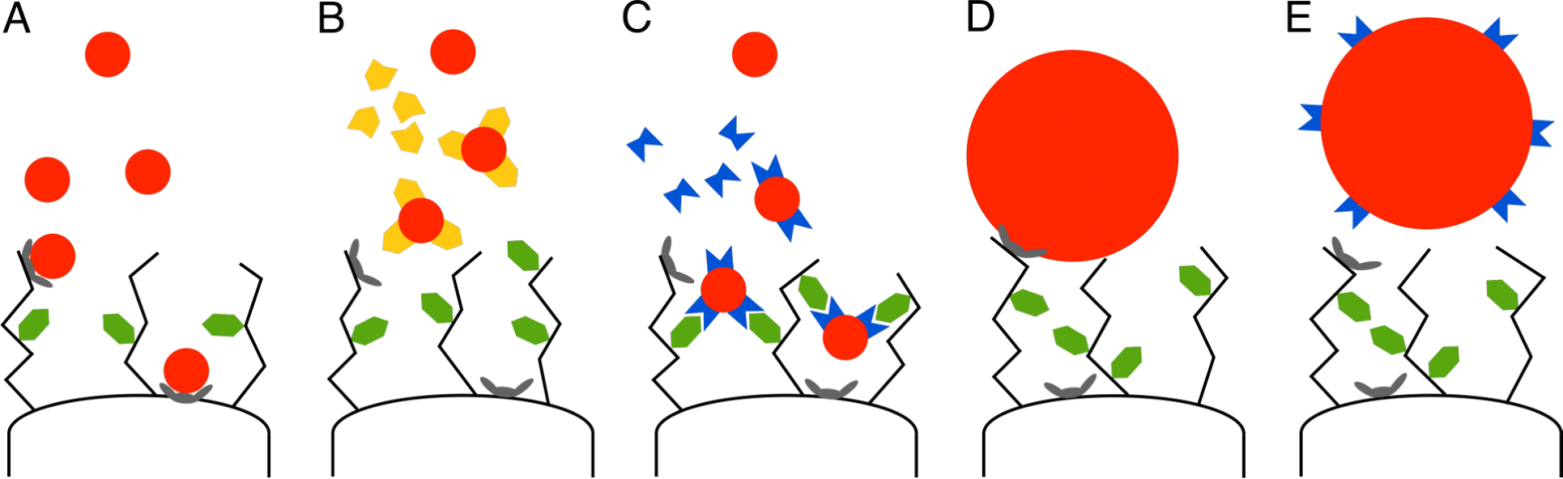
Schematic model of possible interaction mechanisms between differently sized NPs and endothelial cell surface. A: Non-specific binding of “naked” small NPs to outer surface structures and membrane components; B: Decreased non-specific binding of small NPs after “passivation” with proteins; C: Increased binding of small NPs pretreated with luminal gut-constituents to specific attachment sites within the glycocalyx; D: Non-specific binding of “naked” large NPs to outer surface structures of the cells; E: Abolished binding of large NPs pretreated with luminal gut-constituents since particles are too large to penetrate the glycocalyx and reach specific attachment sites.

About the reason for the divergent effects of the different proteinaceous compounds on particle attachment we can only speculate (Figure 5). Without having come into contact with proteins, “naked” NPs may interact non-specifically with surface structures either in the glycocalyx or – if the particles are small enough to penetrate this dense meshwork of glycostructures blanketing the epithelial cells – directly on the cell membrane. This interaction-type can be abolished by treating the particles with proteins (“passivation” of particle surface). Coating of particles with constituents of the intestinal fluid, on the other hand, may result in more complex attachment mechanisms. The intestinal fluid is a mixture of a variety of substances, it contains, among others, digestive enzymes like trypsin or chymotrypsin and also considerable amounts of immunoglobulin A and mucin [30]. It is known that components of the intestinal fluid, e.g., digestive enzymes from the gut lumen, can be immobilized at the outer edge of the enterocyte surface by attachment to lipoprotein and mucopolysaccharide structures in the glycocalyx. They account for the so-called membrane digestion, a step believed to be relevant to the activity of the alimentary tract [31]. Along this line, it is conceivable that small NPs 20 and 100 nm in size covered by, e.g., digestive enzymes from the intestinal fluid may be captured by respective structures of the glycocalyx. Larger (200 nm) NPs may simply be too bulky to penetrate into the dense glycocalyx mesh and reach the respective attachment sites.

In conclusion, our in vitro data indicate that certain individual proteins, like BSA or casein, are able to “passivate” NP surfaces thereby reducing attachment to cells. In contrast, native intestinal fluid obviously contains components which may promote NP adherence to the cell surface. The different capabilities of proteins to modify particles and interact with surface structures of the cell determine the dimension of the NP-cell contact.

## Experimental

### Chemicals

BSA (fraction V fatty acid free) and meat extract were obtained from Sigma-Aldrich (Taufkirchen, Germany), casein (Hammarsten grade) was from BDH Prolabo (via VWR International GmbH, Darmstadt, Germany). Cell culture medium and supplements were obtained from PAA Laboratories GmbH (Pasching, Austria) and Roche Applied Science (Mannheim, Germany). Native intestinal fluid was harvested from the dissected small intestine of Balb/c mice as described previously [30].

### Cell culture

Human Caco-2 BBe1 cells (ATCC, via LGC Standards, Wesel, Germany) of passage 68–73 were maintained in DMEM, high glucose with sodium pyruvate, supplemented with 4 mM L-glutamine, 10% fetal bovine serum (FBS), 10 µg/mL human transferrin and 1% penicillin–streptomycin solution. The cells were cultured at 37 °C in a humidified atmosphere containing 10% CO_2_ and the medium was changed every 2–3 days.

### Nanoparticles

Carboxylate-modified polystyrene NPs 24 ± 4.0, 100 ± 6.0 and 210 ± 10 nm in size (in this study referred to as 20, 100 and 200 nm) were purchased from Life Technologies (Invitrogen Molecular Probes^®^; Darmstadt, Germany). All three particle types contain a yellow-green fluorescent dye with a fluorescence spectrum corresponding to fluorescein. The fluorescence stability of the NPs in presence of luminal gut-constituents was checked.

### Transmission electron microscopy of Caco-2 cells

Caco-2 cells were seeded at 7.5 × 10^4^ cells/cm^2^ on transwell inserts of 24 well plates (polyester membrane, pore size 0.4 µm; Corning Life Sciences, Amsterdam, Netherlands) and cultured until the cells formed a confluent monolayer and further 7, 14 or 21 days. For transmission electron microscopical analysis, the cells were treated according to the protocol of Bye et al. [32]. They were washed once with 0.1 M sodium cacodylate (pH 7.4) and fixed with a solution containing osmium tetroxide and glutaraldehyde as fixatives (0.5% osmium tetroxide (Polysciences Europe GmbH, Eppelheim, Germany), 2.5% glutaraldehyde, 10 mM potassium hexacyanoferrate(II) (both from Sigma-Aldrich/Fluka, Taufkirchen, Germany), 0.05 M sodium cacodylate (Carl Roth, Karlsruhe, Germany)). After fixation the cells were washed once again with sodium cacodylate, dehydrated over a serial dilution with ethanol and embedded in epoxy resin. Ultrathin sections were stained with uranyl acetate and lead citrate, and examined with a Zeiss EM910 electron microscope; images were taken at 2,500× and 12,500× magnification.

### Nanoparticle adherence experiments

Caco-2 cells were seeded at 7.2 × 10^4^ cells/cm^2^ on 96-well microtiter plates, black with transparent bottom (ibidi GmbH, Martinsried, Germany) and cultured as described above until 14 days after having formed a confluent monolayer. At this point in time, the morphology of differentiated intestinal cells was manifest. The culture medium was replaced by HBSS (Hank’s balanced salt solution, pH 7.4) and the cells were incubated in HBSS at 37 °C for 30 to 40 min. NPs were suspended in HBSS and the individual proteinaceous constituents were added (control: no protein, BSA: 4 mg/mL, casein: 1 mg/mL, meat extract: 2 mg/mL, intestinal fluid: 0.5% (v/v)). After 30 min equilibration, the HBSS was removed from the Caco-2 cells, the respective NPs in the protein suspensions were added and the cells were incubated at 37 °C for one hour. In a second setting of experiments the individual proteinaceous constituents were added to the cells first and incubated for one hour. After this time, the protein solutions were carefully removed and the cells were either once washed with HBSS or directly used further without a washing step. A suspension of the respective NPs in protein free HBSS was added to the cells and incubated for one hour. The following NP amounts per cm^2^ were used in each experiment: 20 nm 5.2 × 10^11^, 100 nm 1.2 × 10^10^, 200 nm 1.1 × 10^9^. The experiments were terminated by washing the cell monolayer three times with Ca- and Mg-containing PBS, pH 7.4 (CM-PBS; PAA Laboratories GmbH).

In addition Caco-2 cells grown on transwell filters (7.2 × 10^4^ cells/cm^2^) for 14 days were incubated with 100 nm NPs (2.7 × 10^10^ particles/cm^2^) and BSA (2 mg/mL) for one hour at 37 °C. The cells were treated in the same way as it was described above for the NPs adherence experiments. After the final washing step the cells were fixed with 3% paraformaldehyde (40 min at rt) and subsequently labeled with the lectin Ricinus communis agglutinin I (RCA I, Vector Laboratories, Burlingame, USA) to visualize the cell surface. Therefore the fixed cells were incubated with 50 mM NH_4_Cl in PBS (15 min at rt), washed again, and incubated with 0.2% gelatin in PBS (60 min at rt) to block nonspecific protein binding sites. The biotinylated lectin RCA I was used in a concentration of 20 µg/mL in 0.2% gelatin/PBS to label the cells over night at 4 °C. Afterwards the cells were incubated with Cy5-conjugated streptavidin (GE Healthcare, Brand Amersham, Pittsburgh, USA), 5 µg/mL in 0.2% gelatin/PBS, for 90 min at rt. Finally, the transwell filters were embedded in Mowiol-Dabco (10% (w/w) Mowiol 4-88, 25% (w/w) glycerol, 2.5% (w/w) 1,4-diazabicyclo[2.2.2]octane, 0.1 M Tris-HCl, pH 8.5) on object slides.

In all binding studies, cell viability was assessed by microscopical inspection of cell morphology after every incubation step; no adverse effects were discernible after addition of NP, meat extract, BSA or casein. Incubation of cells with intestinal fluid (with or without particles) resulted in slight injuries in the cell layer caused by the digestive enzymes of the intestinal fluid. Preliminary tests using different dilutions of intestinal fluid were done to determine the degree of damage. The maximal concentration of intestinal fluid causing minimal formation of holes (0.5% v/v) was chosen for the experiments. At the end of each experiment the cell monolayer was scanned with the Relative Tile experiment setting (see below) in phase contrast mode to confirm its over-all integrity.

### Microscopical analysis and statistics

Microscopic analysis of the NP adherence experiments in the 96-well microtiter plates was performed with the More™ fluorescence microscope system (FEI Life Sciences, formerly TILL Photonics, Gräfelfing, Germany) using the following settings: Oligochrome light source, objective lens 10×, filter set ET480/40×, T495LPXR, ET535/50m (FITC). Single wells were scanned by applying the Relative Tile experiment setting where a tile of 5 by 6 images around a selected position is acquired. The individual tiles were fused to one relative tile by the Live Acquisition Software (version 2.22, FEI Life Sciences Munich GmbH, Germany). The total fluorescence intensity in the relative tile was determined by ImageJ (version 1.46a, NIH, Bethesda, USA). The statistical analysis was performed with Graph Pad Prism (version 5.04, Graph Pad Software, Inc., La Jolla, USA).

The cells on the transwell filters were microscopically examined with the same settings as above, except for the objective lens which was changed to 40× and the additional use of a second filter set (ET620/60x, T660LPXR, ET700/75m; Cy5). With both filter sets (FITC and Cy5) *z*-stacks were recorded over 20 frames with 1 µm *z*-slices by using Live Acquisition Software. After processing the 2D image sets in ImageJ the 3D rendition was performed with Imaris x64 (version 7.4, Bitplane AG, Zurich, Switzerland).
